# The narrow-spectrum anthelmintic oxantel is a potent agonist of a novel acetylcholine receptor subtype in whipworms

**DOI:** 10.1371/journal.ppat.1008982

**Published:** 2021-02-05

**Authors:** Tina V. A. Hansen, Susanna Cirera, Cédric Neveu, Elise Courtot, Claude L. Charvet, Kirstine Calloe, Dan A. Klaerke, Richard J. Martin

**Affiliations:** 1 Department of Veterinary and Animal Sciences, Faculty of Health and Medical Sciences, University of Copenhagen, Frederiksberg C, Denmark; 2 INRAE, Université de Tours, ISP, Nouzilly, France; 3 Department of Biomedical Sciences, College of Veterinary Medicine, Iowa State University, Ames, Iowa, United States of America; McGill University, CANADA

## Abstract

In the absence of efficient alternative strategies, the control of parasitic nematodes, impacting human and animal health, mainly relies on the use of broad-spectrum anthelmintic compounds. Unfortunately, most of these drugs have a limited single-dose efficacy against infections caused by the whipworm, *Trichuris*. These infections are of both human and veterinary importance. However, in contrast to a wide range of parasitic nematode species, the narrow-spectrum anthelmintic oxantel has a high efficacy on *Trichuris spp*. Despite this knowledge, the molecular target(s) of oxantel within *Trichuris* is still unknown. In the distantly related pig roundworm, *Ascaris suum*, oxantel has a small, but significant effect on the recombinant homomeric Nicotine-sensitive ionotropic acetylcholine receptor (*N*-AChR) made up of five ACR-16 subunits. Therefore, we hypothesized that in whipworms, a putative homolog of an ACR-16 subunit, can form a functional oxantel-sensitive receptor. Using the pig whipworm *T*. *suis* as a model, we identified and cloned a novel ACR-16-like subunit and successfully expressed the corresponding homomeric channel in *Xenopus laevis* oocytes. Electrophysiological experiments revealed this receptor to have distinctive pharmacological properties with oxantel acting as a full agonist, hence we refer to the receptor as an *O*-AChR subtype. Pyrantel activated this novel *O*-AChR subtype moderately, whereas classic nicotinic agonists surprisingly resulted in only minor responses. We observed that the expression of the ACR-16-like subunit in the free-living nematode *Caenorhabditis elegans* conferred an increased sensitivity to oxantel of recombinant worms. We demonstrated that the novel *Tsu-*ACR-16-like receptor is indeed a target for oxantel, although other receptors may be involved. These finding brings new insight into the understanding of the high sensitivity of whipworms to oxantel, and highlights the importance of the discovery of additional distinct receptor subunit types within *Trichuris* that can be used as screening tools to evaluate the effect of new synthetic or natural anthelmintic compounds.

## Author summary

The human whipworm, *Trichuris trichiura*, is an intestinal parasitic nematode infecting approximately 289.6 million people globally, primarily children living in developing countries. Chronic *T*. *trichiura* infection may cause dysentery, growth stunting and decreased cognitive performance. Whipworm infections are notoriously difficult to control with most available anthelmintics, including those commonly used in mass drug administration programs. Recent randomised controlled trials with whipworm-infected humans, have reported superior efficacies of oxantel, a classic, narrow-spectrum anthelmintic, developed for the treatment of *Trichuris* infections. Despite this knowledge, the molecular target(s) of oxantel within the whipworm has not been identified. In this study, we used the whipworm from pigs as a model and identified a receptor, which was explored using the *Xenopus* oocyte expression system. We demonstrated that this receptor is highly responsive to oxantel, and therefore a major target of oxantel within *Trichuris*. In addition, we discovered that this receptor-type is distinctive and only present in the ancient group of parasitic nematodes, Clade I, which also includes the important zoonotic parasite *Trichinella*. Our findings, explain the specific mode of action of oxantel and open the way for additional characterization of similar receptor subtypes in other medically or veterinary important parasitic nematodes of Clade I.

## Introduction

The human whipworm, *Trichuris trichiura*, is a Clade I parasitic nematode [1] and one of the Soil Transmitted Helminths (STHs) that is estimated to infect 289.6 million people globally, primarily those living in the tropics and subtropics [2]. Trichuriasis is rarely fatal, but chronically affects the health and nutritional status of the host [3, 4], and is known to be notoriously difficult to treat using current anthelmintic drugs (e.g. albendazole and mebendazole) [5–12]. The extensive use of anthelmintics in livestock has led to widespread anthelmintic resistance (AR) to all the major drug classes [13]. Therefore AR in human parasitic nematodes is a concern where decreased susceptibility to albendazole has already been reported for both *T*. *trichiura* [14] and the human roundworm, *Ascaris lumbricoides* [15].

Oxantel, is a cholinergic agonist [16], and a *m*-oxyphenol analogue of pyrantel which was developed in 1972 [17] and marketed as a veterinary anthelmintic in 1974 for the treatment of *Trichuris* [18]. Early clinical trials reported oxantel to be effective against *T*. *trichiura* infections [19, 20] and recent studies show that oxantel is superior to single-dose albendazole and mebendazole [21, 22], which are currently recommended by the WHO for the control of STHs [23]. Cholinergic agonists [16] exert their effect by paralyzing the worms, which are subsequently killed or expelled from the host [24]. This effect is mediated by nicotinic acetylcholine receptors (nAChRs) [24] that are either heteromeric or homomeric five-subunit ligand-gated ion channels expressed in neuronal, muscle and non-neuronal cell membranes [25, 26]. nAChRs of parasitic nematodes have been separated into different pharmacological subtypes based on their sensitivities to a range of cholinergic anthelmintics. Patch-clamp recordings of muscle cells isolated from the pig roundworm *A*. *suum*, have revealed that their muscle nAChRs are preferentially activated either by levamisole (L), nicotine (N) or bephenium (B), and correspondingly are described as *L*-, *N*-, and *B*- AChRs subtypes [27]. Oxantel is classified as an agonist which is selective for the *N*-AChR subtypes [16]. The *N*-AChR subtypes from the model nematode *Caenorhabditis elegans* and the distantly related pig parasite *A*. *suum* are homomeric receptors made of the ACR-16 subunits [28, 29]. Both of these ACR-16 receptors have a low, but significant sensitivity to oxantel [29, 30].

The high sensitivity of *Trichuris* spp. to oxantel has previously been speculated to be due to an nAChR subtype present in *Trichuris* spp. that differs from nAChRs present in other intestinal parasitic nematodes [16]; we hypothesized that a potential homolog of ACR-16 in *Trichuris* could be a target of oxantel within this species.

Here we describe the functional characterization of a novel AChR subtype from the pig whipworm *T*. *suis* with a high sensitivity to oxantel and distinctive pharmacological properties. This homomeric receptor, referred to as an *O*-AChR subtype, is made of a divergent subunit specific to Clade I nematode species that is only distantly related to ACR-16 from nematode species belonging to other clades. Our results provide new insights about the mode of action of oxantel, its high efficacy on whipworms, and the divergent anthelmintic sensitivity of whipworms.

## Results

### Identification of *T*. *suis* sequences related to the ACR-16 group

**Fig 1:**
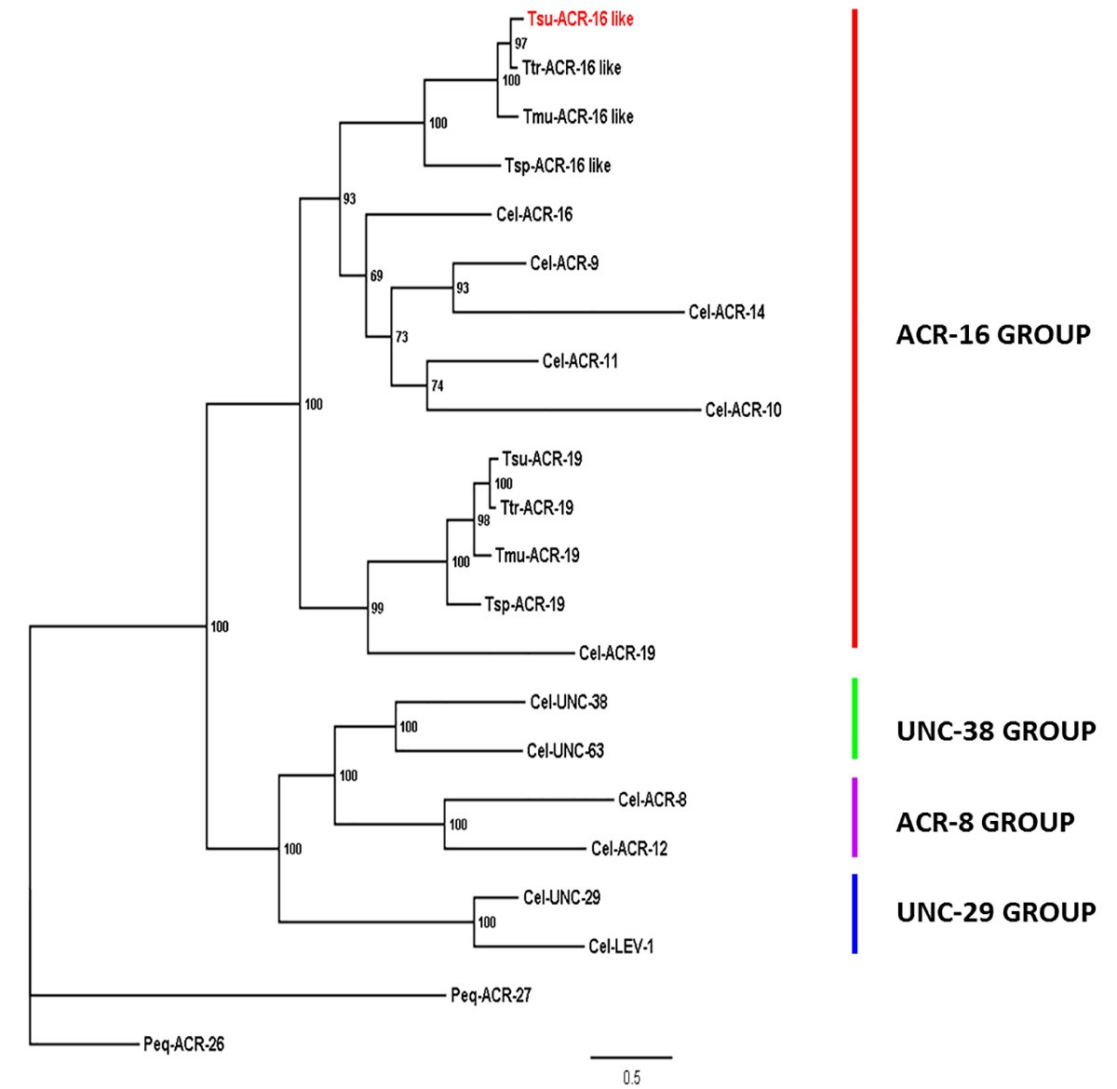
Maximum likelihood tree showing relationships of the ACR-16 related acetylcholine receptor (nAChR) subunits from *Trichuris* spp., with other *C*. *elegans and T*. *spiralis* nAChR subunits. The tree was built upon an alignment of nAChR subunit deduced amino-acid sequences. The tree was rooted with the *Parascaris equorum* ACR-26 and ACR-27 sequences that are absent from *C*. *elegans* and clade I nematode species [34]. Scale bar represents the number of substitutions per site. Bootstrap values are indicated on branches. Accession numbers for sequences used in the phylogenetic analysis are provided in the Material and Methods section. *C*. *elegans* nAChR subunit groups are named as proposed by Mongan *et al*. [31], *Cel*, *Tsu*, *Ttr*, *Tmu*, *Tsp* and *Peq* refer to *Caenorhabditis elegans*, *Trichuris suis*, *Trichuris trichiura*, *Trichuris muris* and *Parascaris equorum*, respectively.

Using the *C*. *elegans* ACR-16 deduced amino-acid sequences as a query, tBLASTn search against *T*. *suis*, *T*. *muris* and *T*. *trichiura* genomic data available in WormBase-ParaSite (version WBPS14; http://parasite.wormbase.org/) allowed the identification of two distinct hits for each species sharing identities ranging from 46% to 47% with the *C*. *elegans* sequence. Subsequently, a second tBLASTn search against nematodes genomic data available at the NCBI was performed with the retrieved *Trichuris spp*. sequences. Homologies could be identified with either of the *acr-16* or *acr-19* genes from nematode species representative from the nematoda phylum. Using a panel of representative *C*. *elegans* nAChRs subunits as references, a phylogenetic analysis including the *Trichuris* spp. and their putative homologs in the closely related species *Trichinella spiralis* was carried out (Fig 1). *Trichuris* spp. sequences were found to form two distinct clusters. The first one presented a clear orthologous relationship with ACR-19, the second one clustered apart from the other subunits and belonged to the ACR-16 group [31]. An additional analysis, including other related sequences from nematode species representative from the different clades of the *nematoda* phylum (S1 Fig) further confirmed that Clade I nematode species (including *Trichuris* spp.) possess a divergent group of AChR subunit related to the ACR-16 subunit. Consequently, the corresponding sequences from Clade I nematode species were named ACR-16-like.

### Molecular cloning of the *Tsu*-acr-16-like coding sequence

**Fig 2:**
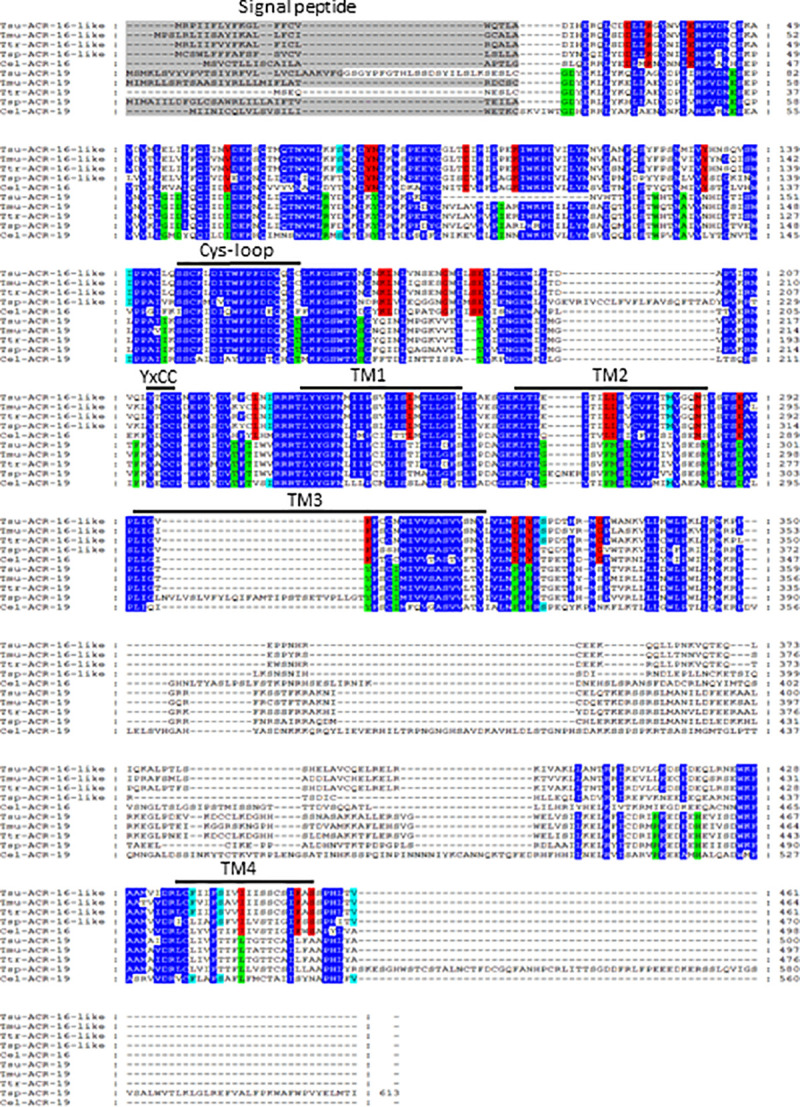
Amino acid alignment of ACR-16-(like) and ACR-19 subunit sequences from the Clade I parasitic nematodes *Trichuris suis*, *T*. *trichiura*, *T*. *muris*, *Trichinella spiralis* and *Caenorhabditis elegans*. Predicted signal peptide sequences are shaded in grey, the Cys-loop, the transmembrane regions (TM1-TM4), and the YxCC motif that characterize an α-subunit are indicated above the sequences. Conserved amino acids between ACR-16-(like) and ACR-19 sequences (dark blue), conserved amino acids between all ACR-16-(like) sequences (red), conserved amino acid between all ACR-19 sequences (light green), conserved amino acid between ACR-16-like sequences of Clade I parasitic nematodes and ACR-19 of *C*. *elegans* (light blue).

In the present study, based on the current knowledge of the original mode of action of oxantel [16, 29], we hypothesized that the divergent ACR-16-like from *Trichuris* spp. could represent a preferential target for this narrow-spectrum anthelmintic. Using *T*. *suis* as a model, we cloned its *acr-16-like* full-length cDNA sequence as a matter of priority and deposited the sequence in GenBank under the accession number MT386096. During PCR amplification for the cloning, we observed several band sizes and tried unsuccessfully to clone all of them. Therefore, it is possibly that there are at least 2 other isoforms of the *Tsu-*ACR-16-like subunits. An alignment of the *Tsu*-ACR-16-like sequence with its closely related counterparts from *T*. *muris*, *T*. *trichiura*, *T*. *spiralis* and *C*. *elegans* is provided in Fig 2. The *Tsu-*ACR-16-like subunit was found to share typical features of nAChR subunits including a predicted signal peptide, a Cys-loop motif, four transmembrane regions (TM1-TM4), and the YxCC motif which characterize an α-type nAChR receptor subunit.

### *Tsu*-ACR-16-like subunits form a functional homomeric receptor when co-expressed with the ancillary protein RIC-3 in *Xenopus laevis* oocytes

Previous studies have shown that *Cel*-ACR-16 and *Asu-*ACR-16 can form functional homomeric receptors when expressed in *X*. *laevis* oocytes with the ancillary protein RIC-3 [29, 32]. Thus, we explored the requirement of RIC*-*3, and compared *Tsu*-ACR-16-like subunit expression using either the RIC-3 from *Xenopus laevis* (*Xla*-RIC-3), or RIC-3 from various nematode species to reconstitute a functional AChR in *X*. *laevis* oocytes. *Xenopus laevis* oocytes were micro injected with *Tsu-acr-16-like* cRNA in combination with *Xla-ric-*3, *Asu-ric*-3, *Haemonchus contortus* ric-3 isoform 1 (*Hco-ric*-3.1), or *Caenorhabditis elegans* ric-3 (*Cel-ric*-3). *Tsu-acr-16-like* cRNA or *Asu-ric*-3 cRNA alone as well as non-injected oocytes were used as controls.

**Fig 3:**
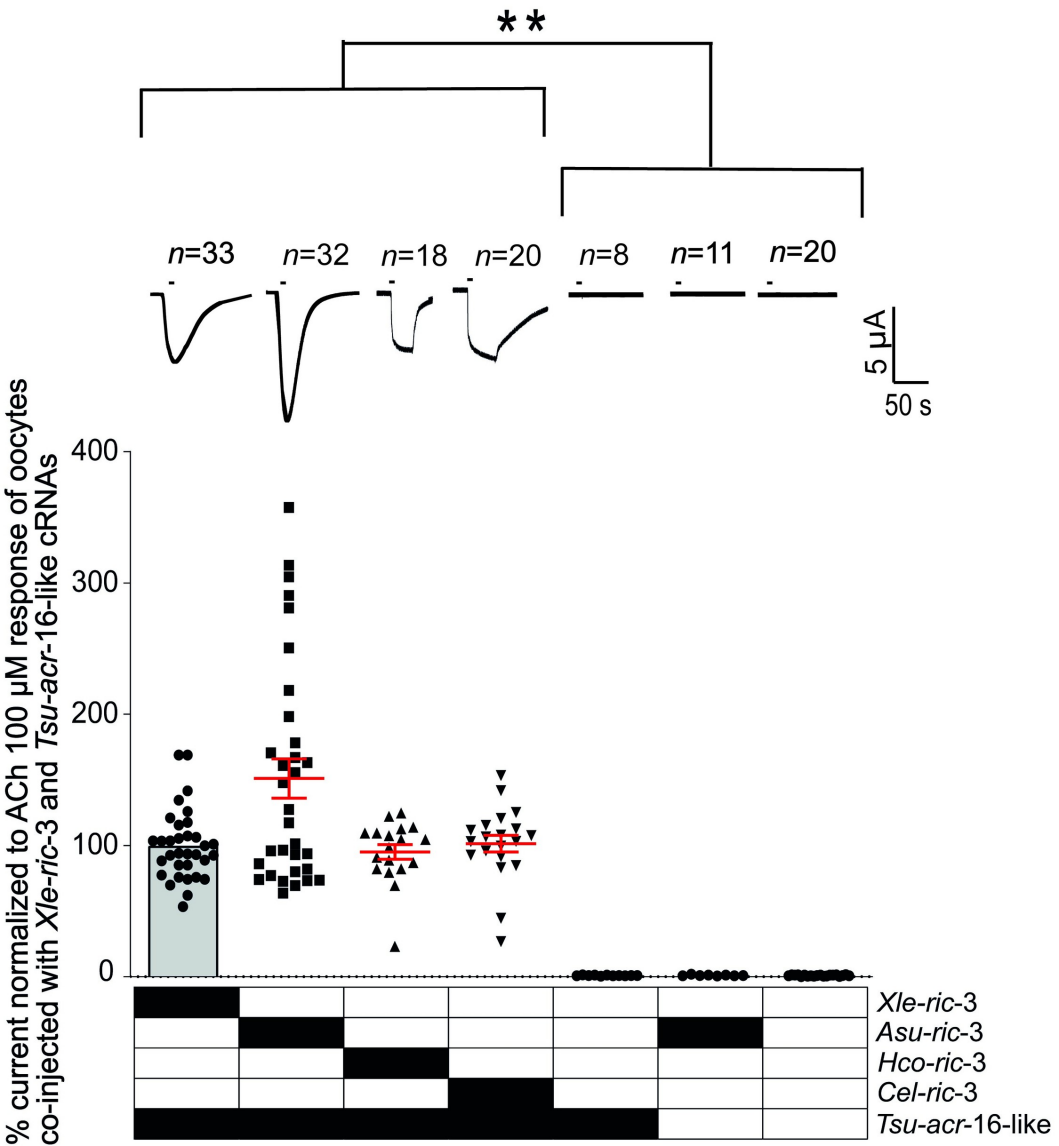
Effect of the ancillary protein Resistance-to-cholinesterase (RIC-3) from *Xenopus laevis* (*Xla-*RIC-3), *Ascaris suum* (*Asu-*RIC-3), *Haemonchus contortus* isoform 1 (*Hco*-RIC-3.1), *Caenorhabditis elegans* (*Cel*-RIC-3) on the functional expression of the ACR-16-like nAChR from *Trichuris suis* (*Tsu-*ACR-16-like receptor). Representative sample traces of inward current in response to 100 μM ACh are shown together with a scatter dot plot presenting the relative currents (mean ± SEM). Non-injected oocytes and oocytes injected with *Tsu-acr-16-like*- or *Asu-ric-3* cRNA alone did not respond to 100 μM ACh which was significantly different from oocytes co-injected with *Tsu-acr-16-like* and either of the tested ric-3 cRNAs (**P < 0.01). The relative currents of oocytes co-injected with *Tsu-acr-16-like* and either of the tested ric-3 cRNAs were not significantly different (*P* > 0.9) as indicated, Kruskal-Walis Test.

The combination of *Tsu-acr-16-like* with any of the ric-3 cRNAs led to robust expression of functional receptors responding to 100 μM acetylcholine (ACh), which elicited inward currents in the μA range (Fig 3). The largest relative currents were measured in oocytes co-injected with *Tsu-acr-16-like* and *Asu-ric*-3 cRNA, however, no statistically significant differences (*P* > 0.9) were found between the effect of RIC-3 from *X*. *laevis* and nematodes of Clade III (*A*. *suum*) and Clade V (*C*. *elegans* and *H*. *contortus*). Non-injected oocytes, or oocytes injected with either *Tsu-acr-16-like* or *Asu-ric*-3 cRNA alone, did not respond to 100 μM ACh, highlighting the need of RIC-3 for the functional expression of the homomeric *Tsu*-ACR-16-like receptor. Representative traces of the inward currents for each injection type, and a bar chart presenting their mean ± SEM normalized values are shown in Fig 3. In addition, the results showed no significant difference between the reversal potentials observed in 1 mM (9.7 +/- 4.3 mV) and 10 mM (10.2 +/- 3.4 mV) external Ca^2+^ suggesting a low Ca^2+^ permeability for the receptor Based on these results, all subsequent recordings were performed on oocytes co-injected with *Tsu-acr-16-like* and *Asu-ric*-3 cRNAs.

### Oxantel is a potent agonist on the *Tsu*-ACR-16-like receptor

**Fig 4:**
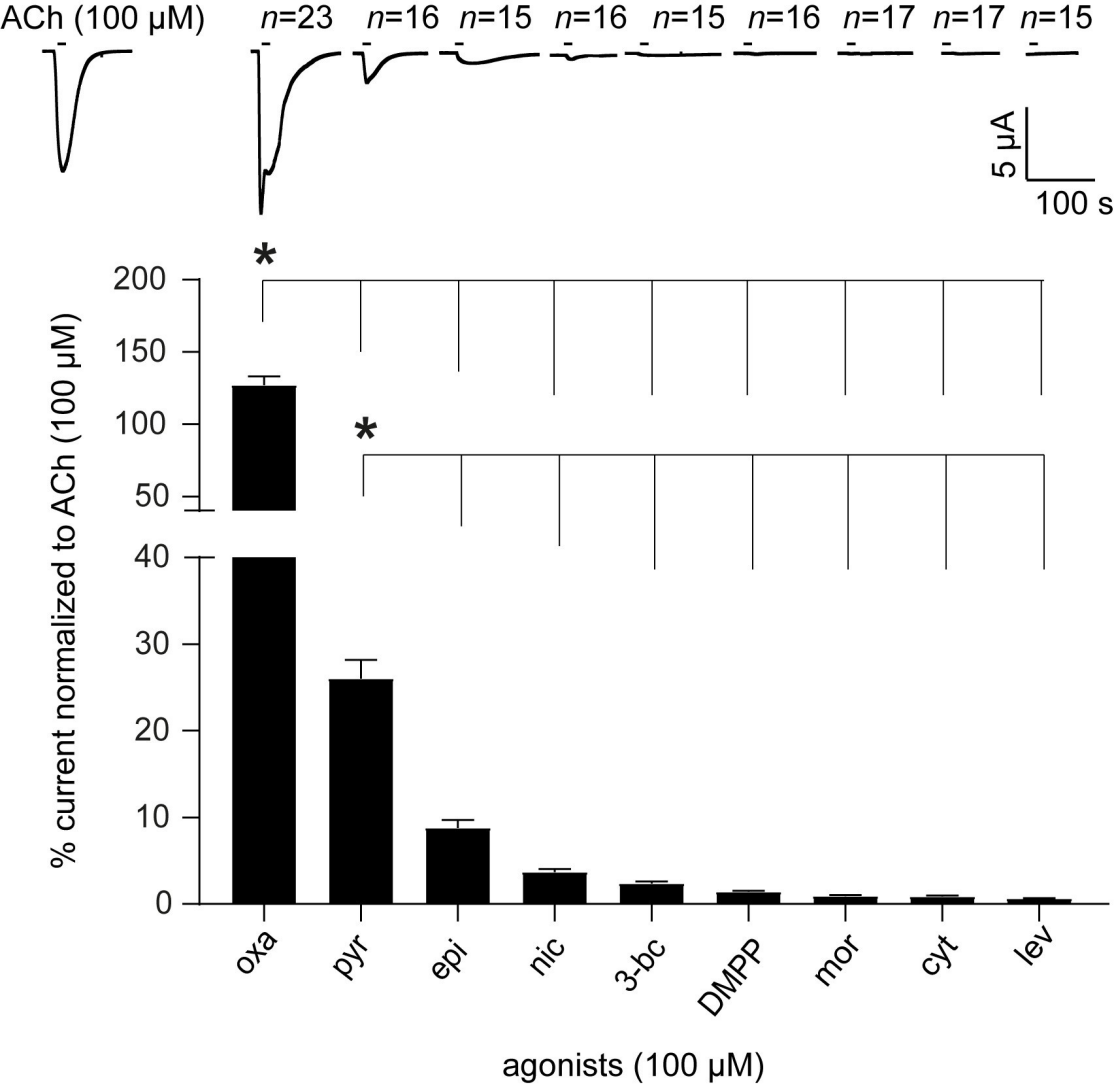
The effect of 9 agonists on *Tsu-*ACR-16-like receptor. Representative sample traces and a bar chart (mean ± SEM) show the rank order potency series of 4 cholinergic anthelmintics: oxantel (oxa), pyrantel (pyr), morantel (mor), levamisole (lev) and 5 nAChR agonists: epibatidine (epi), nicotine (nic), 3-bromocytisine (3-bc), dimethylphenylpiperazinium (DMPP) and cytisine (cyt). *P* < 0.05; significantly different as indicated; Turkey’s multiple comparison test.

To explore the effect of oxantel and perform a detailed pharmacological characterization of the *Tsu-*ACR-16-like receptor, we used 4 cholinergic anthelmintics (i.e. oxantel, pyrantel, morantel and levamisole) and 5 nAChR agonists (i.e. epibatidine, nicotine, 3-bromocytisine, DMPP and cytisine). Fig 4 shows the rank order potency series of these drugs, representative traces of the inward currents induced by each of them, the number of oocytes (*n*) used for each agonist, along with a bar chart presenting the normalized mean ± SEM for each drug group. Oxantel was the most potent test agonist on the *Tsu-*ACR-16-like receptor and induced a current response in the same range as the control response of 100 μM ACh. Pyrantel also induced a relatively high current response, however; the nicotinic agonists: epibatidine, nicotine, 3-bromocytisine, DMPP and cytisine as well as the cholinergic anthelmintics, morantel and levamisole, were the least potent. The rank order potency series for the agonist drugs on the *Tsu-*ACR-16-like receptor when normalized to control oocytes exposed to 100 μM ACh was: oxantel ~ ACh >>> pyrantel >>> epibatidine > nicotine ~ 3-bromocytisine ~ DMPP ~ morantel ~ cytisine ~ levamisole. Taken together, these observations provide strong evidence that the *Tsu*-ACR-16-like receptor represents the preferential molecular target for oxantel. Interestingly, when exposed to 100 μM ACh for 1–3 min the *Tsu-*ACR-16-like receptor did not show fast-desensitization kinetics (S2 Fig) which is a distinctive characteristic of the *N*-AChR of nematode such as *Asu*-ACR-16 [29] and the ACR-16 from *Parascaris equorum* (*Peq*-ACR-16) [33].

### Dose-response curve of oxantel and pyrantel

**Fig 5:**
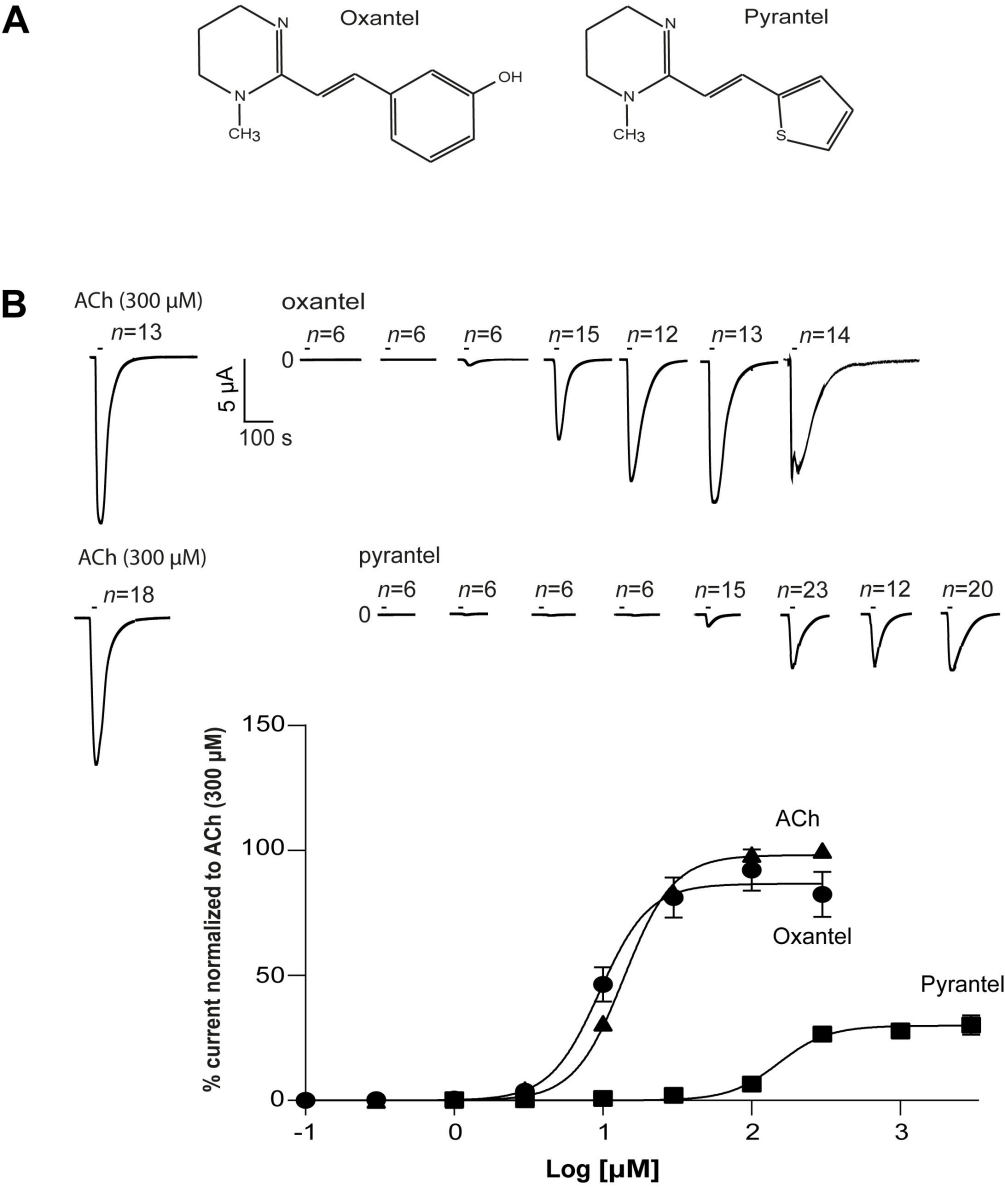
A: Chemical structure of oxantel and pyrantel. Oxantel: free drawing after https://pubchem.ncbi.nlm.nih.gov/compound/oxantel#section=2D-Structure. Pyrantel: free drawing after https://pubchem.ncbi.nlm.nih.gov/compound/pyrantel#section=2D-Structure **B:** Dose-response curves for oxantel (oxa), pyrantel (pyr) and acetylcholine (ACh). The current response on *Tsu-*ACR-16-like receptor is normalised to current responses induced by 300 μM ACh and given as mean ± SEM. The EC_50_ ± SD values were 9.48 ± 1.15 μM for oxa, 152.7 ± 1.20 μM for pyr and 14.5 ± 1.03 for ACh, the relative maximum current responses, *I*_max_ were 86.85 ± 4.63% and 29.41 ± 1.95%, and the Hill slope, *n*_H,_ 2.51 ± 1.30, 3.13 ± 1.07 and 2.14 ± 0.1 for oxa, pyr and ACh respectively.

Oxantel and pyrantel have similar chemical structures (Fig 5A), but their potencies on the *Tsu-*ACR-16-like receptor were found to be significantly different (Fig 4). We performed a dose-response study on oxantel, pyrantel and ACh to determine their *EC*_*50*_ values, their relative maximum current responses, *I*_max,_ and their Hill slopes, *n*_H._ The mean current response of positive control oocytes exposed to 300 μM ACh was used for normalization. Fig 5B shows representative traces and dose-response relationships of the normalized inward currents (mean ± SEM) induced by different concentrations of oxantel, pyrantel and ACh. The resulting *EC*_*50*_ ± SE for oxantel (9.49 ± 1.13 μM) was much lower than that of pyrantel (148.5 ± 1.19 μM, *P* < 0.001) and slightly lower than that of ACh (14.5 ± 1.03). The relative maximum current response, *I*_*max*_ (mean ± SE) was significantly larger for oxantel (86.85 ± 4.63%) than pyrantel (29.41 ± 1.95%, *P* = 0.003), whereas no significant difference was found between the Hill slopes, *n*_H_ (mean ± SE), of oxantel (2.51 ± 1.31), pyrantel (3.13 ± 1.07) and ACh (2.14 ± 0.1).

### Antagonists

**Fig 6:**
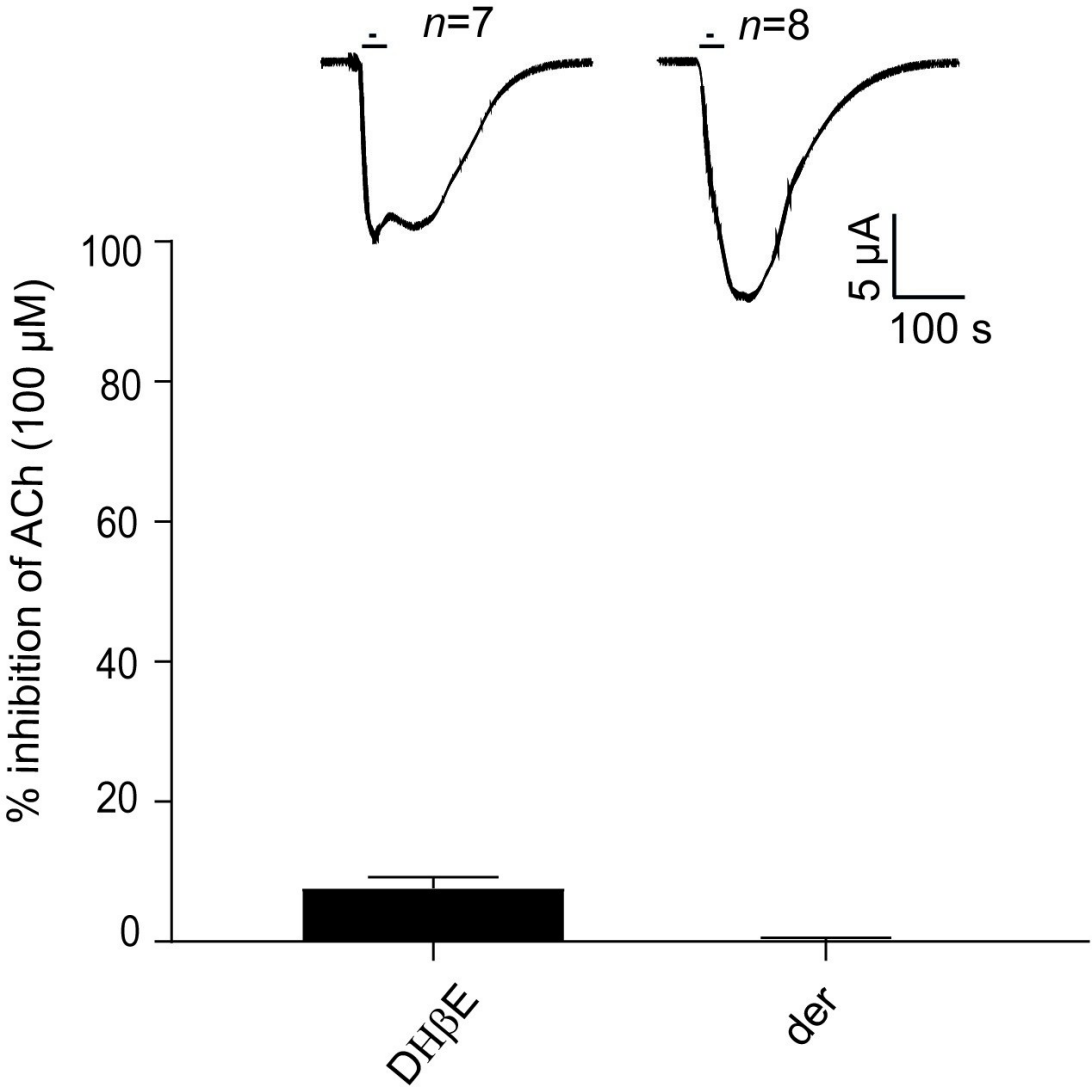
Effect of the antagonists: dihydro-β-erythroidine (DHβE) and derquantel (der) on *Tsu-*ACR-16-like receptor mediated 100 μM ACh current response. Results are given as normalized mean ± SEM inhibition of the initial current response of 100 μM ACh. DHβE produced an almost insignificant block of the *Tsu-*ACR-16-like receptor mediated ACh response (i.e. 7.60 ± 1.61%) and no effect was observed for der (0.16 ± 1.61%).

**Fig 7:**
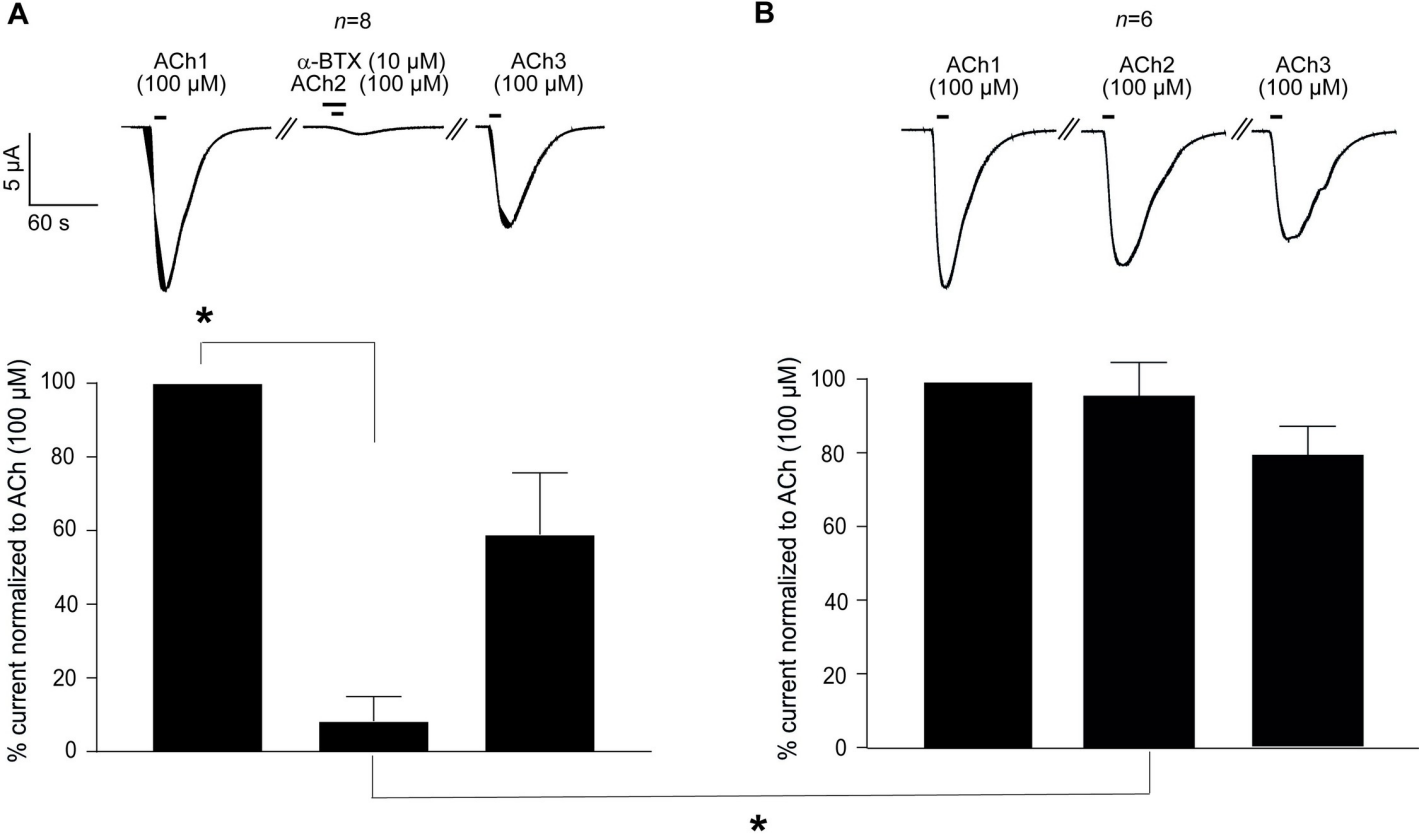
**A and B:** Effect of the antagonists α-bungarotoxin (α-BTX) on *Tsu-*ACR-16-like receptor mediated 100 μM ACh current response. The first ACh current response (ACh1) is set to 100 for both test- (Fig 7A) and control oocytes (Fig 7B), and subsequent current responses are given as normalized mean ± SEM inhibition of ACh1. *P* < 0.05; significantly different as indicated, Dunnett’s test.

To further characterize the pharmacology of *Tsu-*ACR-16-like receptor, we tested 3 selected antagonists: dihydro-β-erythroidine (DHβE), α-bungarotoxin (α-BTX) and the anthelmintic, derquantel. Fig 6 shows the effect of 10 μM DHβE and 10 μM derquantel on the *Tsu-*ACR-16-like receptor along with representative current responses. The initial ACh (100 μM) current response of each oocyte was used for normalization, to measure the reduced current responses in the presence of the antagonists. For DHβE, the mean ± SEM inhibition was very small (i.e. 7.60 ± 1.6%) and no inhibition was observed for derquantel (0.16 ± 1.6%). The effect of α-BTX is given in Fig 7A which shows the response-inhibition of the *Tsu-*ACR-16-like receptor to 100 μM ACh when 10 μM α-BTX is applied 10 s before the second application of 100 μM ACh (test oocytes). Fig 7B shows the effect of the *Tsu-*ACR-16-like receptor when exposed to repetitive applications of 100 μM ACh, only (control oocytes). The first current response of 100 μM ACh (ACh1) was used for normalization. Representative current traces along with a bar chart presenting the normalized mean ± SEM of the second and third drug application of *Tsu-*ACR-16-like receptor expressing- and un-injected control oocytes are given in Fig 7A and 7B. The 10 μM α-BTX significantly (*P* < 0.0001) reduced the current response of ACh to 8.3 ± 2.4% of the *Tsu-*ACR-16-like receptor expressing oocytes (Fig 7A). This reduction was not observed in the control oocytes (Fig 7B), thus, when α-BTX was applied 10 s before the second application of ACh, the current response was significantly reduced in test oocytes as compared to control oocytes (Fig 7A and 7B, *P* < 0.0001).

### The expression of the *Tsu*-ACR-16-like subunit in *Caenorhabditis elegans* increases the sensitivity to oxantel

The heterologous expression of parasitic nematode genes in *C*. *elegans* has successfully been used to decipher the drug sensitivity of parasitic nAChR-subunits [34–36]. To investigate the oxantel sensitivity of the *Tsu-ACR-16*-like receptor, we generated three recombinant *C*. *elegans* lines expressing the *Tsu*-ACR-16 like receptor under the control of the *C*. *elegans myo3* body wall muscular promotor. Wildtype (wt) *C*. *elegans* (named N2) have previously been described to be relative unaffected by oxantel at a concentration of 1 mM [37], thus, ideally suited to investigate the oxantel sensitivity of the recombinant worms.

**Fig 8:**
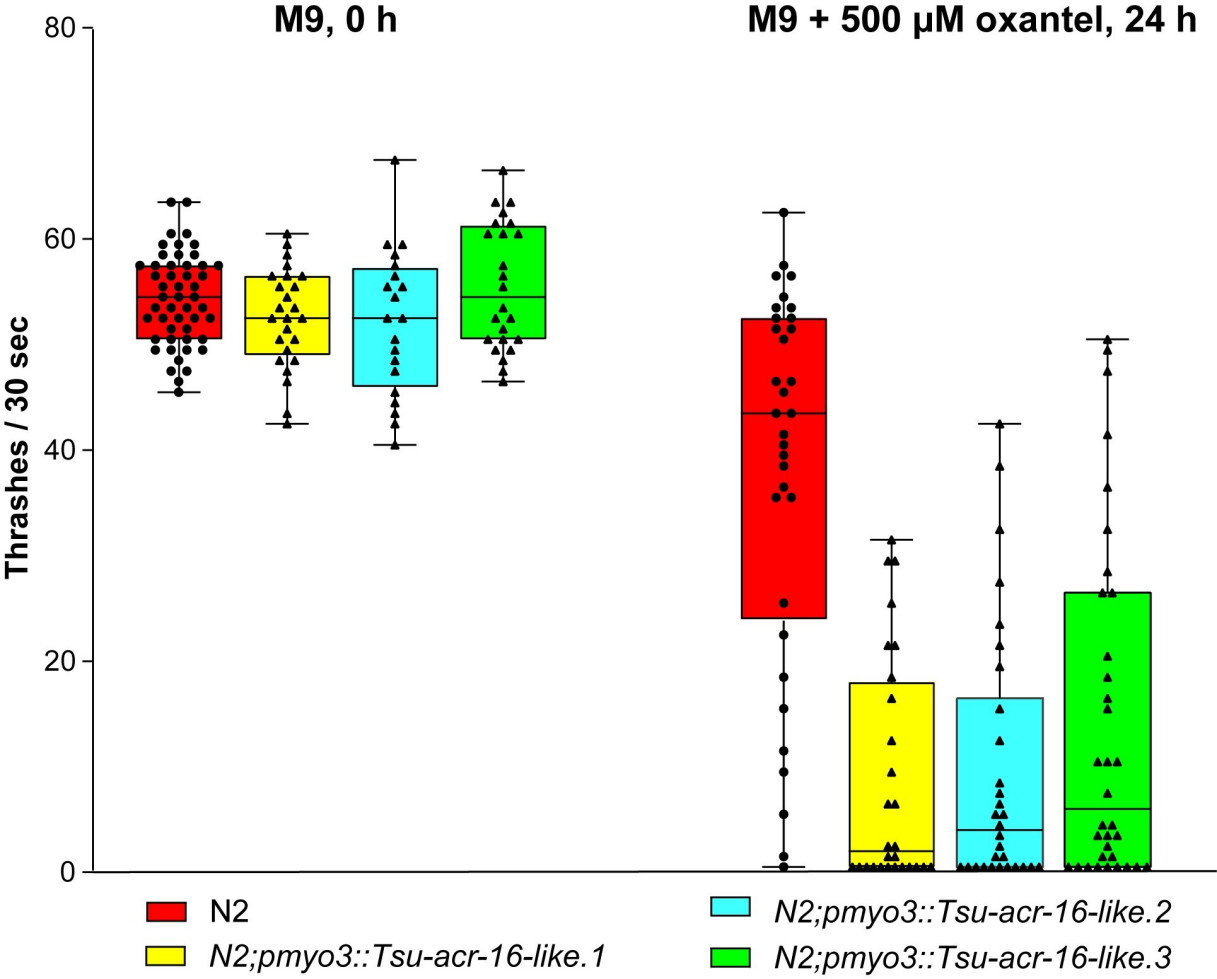
Heterologous expression of *Tsu*-ACR-16-like receptor in *Caenohabditis elegans* confer oxantel sensitivity to the recombinant worms Boxplot depicts number of thrashes/30 sec of worms in M9 media prior to incubation (0 h) and after 24 h incubation with (+) 500 μM oxantel. The number of thrashes were significantly higher for wildtype *C*. *elegans*; N2 (red, *n* = 34) than each of the recombinant *C*. *elegans N2;pmyo3*::*Tsu-acr-16-like* lines: line 1 (yellow, *P* <0.0001, *n* = 28), line 2 (blue, *P* <0.0001, *n* = 30), line 3 (green, *P* <10^−4^, *n* = 34) when incubated in M9 media with 500 μM oxantel.

The effect of 500 μM oxantel on wt N2 and recombinant worms for 24 h is shown in Fig 8 and S1 Video. First, we performed a thrashing assay in M9 medium to determine if the overexpression of *pmyo3*::*Tsu-acr-16-like* receptor had an impact on the basal motility. We observed no significant difference between each line (*P* = 0.25). Second, to investigate the oxantel sensitivity, we performed a thrashing assay after 24 hours of exposure to 500 μM oxantel. Strikingly, we observed a significant difference in the motility between wt N2 and each of the recombinant lines (*P* < 0.001). Indeed, the motility of the recombinant lines was on average inhibited by 80%, whereas the motility of wt N2 worms was only decreased by 30%. As control, we determined the motility of all lines after 24 hours in M9, only, and no significant difference was noticed between neither of the lines (S3 Fig). These results strongly suggests that the *Tsu*-acr-16-like subunit indeed has the ability to confer oxantel sensitivity to *C*. *elegans*, and support that the *Tsu-acr-16-like* receptor is a major target of oxantel within *T*. *suis*. Therefore, the *Tsu-*ACR-16-like receptor will be henceforth referred to as the Oxantel-sensitive AChR (*O*-AChR).

## Discussion

In the present study, we report the identification and the functional expression of the *Tsu-*ACR-16-like receptor, a novel AChR subtype corresponding to the first specific drug target for oxantel to be reported in any nematode species. In reference to the previously reported *L*-AChR, *N*-AChR, and *M*-AChR (respectively for Levamisole-sensitive, Nicotine-sensitive and Morantel- sensitive–AChR subtypes), we named the novel oxantel-sensitive AChR subtype: the *O*-AChR.

### The O-AChR is a novel receptor subtype specific to Clade I nematode species with original pharmacological properties

Use of screens for *C*. *elegans* mutants that survive exposure to the broad-spectrum anthelmintics provided a means to decipher their molecular targets in a wide range of nematode species. However, this approach was not helpful for oxantel because the *C*. *elegans* is insensitive to this drug [37]. The weak, but measurable activity of oxantel on recombinant *N*-AChR from *C*. *elegans* and *A*. *suum*, supported the hypothesis that a putative ACR-16 homologs in *Trichuris* species could be involved in an oxantel-sensitive receptor. Williamson et al. [38] reported that only two members from the ACR-16 group could be identified in the genomic data from the Clade I species *T*. *spiralis*. In agreement with this finding, our search for ACR-16 homologs only retrieved two sequences in each of the *Trichuris* species investigated in the present work. The first one corresponded to the highly conserved AChR subunit encoded by the *acr-19* gene; the second one corresponded to a highly divergent subunit specific to Clade I nematode species designated as ACR-16-like.

When co-expressed in the *X*. *laevis* oocytes with the ancillary protein RIC-3, the ACR-16-like subunits from *T*. *suis* formed a functional homomeric channel (*O*-AChR) with unexpected pharmacological properties. Indeed, this receptor was highly sensitive to oxantel which is in contrast to the *Asu-*ACR-16 for which a low agonist effect of oxantel has been reported (i.e. <10% of the control ACh current) [29] and to the *Cel-*ACR-16 on which oxantel has an antagonistic effect [30]. Likewise, pyrantel had a relatively high effect on the *Tsu-O*-AChR, whereas pyrantel had no agonist effect on *Asu-*ACR-16 [29] or *Cel-*ACR-16, but in contrast, showed an antagonistic effect on the latter, which was ascribed to pyrantel acting as an open channel blocker [28]. In accordance with this assumption, patch-clamp recording studies from isolated *A*. *suum* muscle cells, show that both oxantel and pyrantel act as agonists and open channel blockers [39, 40]. Another surprising difference between the *Tsu-O*-AChR and the ACR-16 receptors from *A*. *suum*, *C*. *elegans* and *P*. *equorum* is the lack of sensitivity to nicotinic agonists [28, 29, 33]. Since oxantel has been characterized as an agonist selective for the *N*-subtypes of the ionotropic AChRs [16] which include the ACR-16 receptors, we expected the *Tsu-O*-AChR receptor to be highly sensitive to nicotine, cytisine, 3-bromocytisine, epibatidine and DMPP, but only small current responses were observed using these agonists. Another feature of the *Tsu-O*-AChR receptor is its slow desensitization kinetics, which contrasts with the faster desensitization of the *N*-AChR from *C*. *elegans* [41], *A*. *suum* [29] and *P*. *equorum* [33].

Interestingly, we also showed that the antagonist, α-BTX had a potent inhibitory effect on the ACh induced current responses of the *Tsu-O*-AChR whereas *A*. *suum* [29] and *C*. *elegans N*-AChR [28] are nearly insensitive to α-BTX. We point out however, that α-BTX only induced a strong inhibitory effect when α-BTX was applied 10 s before the application of ACh suggesting a slow association time of α-BTX. The *Tsu-O*-AChR was virtually insensitive to DHβE and insensitive to derquantel. This also contrasts with the *Asu*-*N*-AChR which is moderately sensitive to DHβE (~ 65% inhibition) and derquantel (~ 60% inhibition) [29] and the *Cel*-*N*-AChR which is highly sensitive to DHβE [28].

Taken together, these results strongly support our hypothesis that *O*-AChR and *N*-AChR represent two distinct classes of ionotropic AChR. Despite the important differences, it is noteworthy that there are also similarities between the *O*- and *N*-AChR: both subtypes are insensitive towards the anthelmintic drugs levamisole or morantel [27–29].

### Sensitivity to oxantel and pyrantel

Small changes in structure of acetylcholine agonists can have large effects on the selectivity and affinity of nicotinic agonists [42]. Perhaps it is not surprising that pyrantel which was modified by replacing the 2-thiophene moiety with a *m*-oxyphenol group [17] to produce oxantel has a different pharmacology. Thus, the anthelmintic spectrum of oxantel and pyrantel is very different. Pyrantel is a broad-spectrum anthelmintic with no effect on adult *Trichuris* [17] whereas oxantel is a narrow-spectrum anthelmintic with a potent and selective effect on adult *Trichuris* [19–22]. This spectrum difference has previously raised the question whether a cholinergic receptor subtype present in *Trichuris* spp. are different from other intestinal nematode parasites [16], which indeed is now strongly supported by our results. In addition, it is remarkable that a recent *in silico* ligand binding analysis of the extracellular domains of ACR-16 from *Ancylostoma caninum*, *Necator americanus*, *C*. *elegans* and *T*. *muris* predicted oxantel to bind to ACR-16 of *T*. *muris* (*Tmu*-ACR-16-like) with high affinity [43]. Presuming that the ACR-16 of *T*. *muris* and *T*.*suis* have similar pharmacological profiles, our results indeed support these docking simulations.

In our study, we observed that oxantel, but not pyrantel, in some recordings induced a “noisy” channel activation at high concentration (i.e. 300 μM), an initial increase in current on washout, and a slightly lower response to 300 μM than 100 μM. These observations suggest oxantel to act as an open channel blocker on the *Tsu-*ACR-16-like receptor and is in accordance with what previously described for *A*. *suum* [39].

### The *Tsu*-acr-16-like receptor confers oxantel sensitivity to *Caenorhabditis elegans*

To confirm the sensitivity of the *Tsu*-ACR16-like receptor to oxantel, we heterologous expressed *Tsu*-ACR-16 like receptor in *C*. *elegans*. We observed a decreased motility of the recombinant worms when exposed to oxantel for 24 h, confirming the sensitivity of the *Tsu*-ACR16-like receptor to oxantel. Expression of the *Tsu*-ACR16-like receptor was under the control of the *myo3* body wall muscular promotor of *C*. *elegans*. This promotor was chosen as oxantel has shown a paralyzing effect on *Trichuris* spp. *in vitro* [44] suggesting a muscular location of the oxantel target within this genus. However, the tissue location of the *Tsu*-ACR-16-like receptor remains to be elucidated. Our results show the ability of *C*. *elegans* to express AChR-subunits from a Clade I parasitic nematode in addition to Clade III and V (i.e. *P*. *equorum* and *H*. *contortus*) [36], thus confirming the *C*. *elegans* expression system to be a valuable screening tool.

### Sensitivity of *Trichuris* spp. to cholinergic drugs *in vitro*

Although *T*. *suis* and *T*. *muris* are different species [45], both are used as models for *T*. *trichiura*. *Trichuris muris* has been extensively used to evaluate the sensitivity of the worms to several cholinergic anthelmintics *in vitro* [8, 44, 46]. Based on motility scores, the inhibitory concentrations (IC_50_) have been reported for fourth-stage larvae (L4) incubated in oxantel pamoate (IC_50_ = 2.35 μg/mL equal to 3.9 μM) [44], third-stage larvae (L3) and adult worms incubated in pyrantel pamoate (IC_50_ = 95.5 and 34.1 μg/mL, respectively, equal to161 and 57 μM), levamisole (IC_50_ = 33.1 and 16.5 μg/mL, respectively, equal to 162 and 80.8 μM) and monepantel (IC_50_ = 78.7 μg/mL for L3, equal to166 μM) for which the latter, adult worms are not sensitive [8, 46]. Thus, oxantel pamoate has been shown to be superior to pyrantel, levamisole and monepantel for *T*. *muris in vitro*.

### Pharmacokinetics of different cholinergic anthelmintics and efficacy against *Trichuris spp*

Recall that the *Trichuris* spp. are located in the large intestines of humans and domestic animals so that concentration of anthelmintics seen by these parasites will be affected by absorption of the anthelmintic preparation along the intestine as well as the plasma concentration.

The high efficacy of oxantel pamoate could be due to the pharmacokinetic profile of the drug, as the pamoate salt limits the absorption of oxantel, increasing the concentration in the digestive tract [18, 47]. In this context it is interesting to compare pyrantel and oxantel as these are similar in molecular structure, are both therapeutically used as pamoate salts but have very different anthelmintic properties against *Trichuris* spp. *in vitro* and *in vivo* (i.e. pyrantel is not effective against *Trichuris* spp. *in vivo* and *in vitro*). Bjørn et al. [48] have shown that pyrantel pamoate stays mostly in the intestinal lumen of pigs and has a relatively high efficacy against *Oesophagostomum dentatum*, a strongyle species that has the same habitat as *Trichuris* spp. in the large intestine [49–52]. The low efficacy of pyrantel pamoate against *Trichuris* spp. is therefore not caused by an absence of intestinal bioavailability of pyrantel pamoate at the site of *Trichuris* spp., but more due to a lower impact of pyrantel on the molecular drug-target within the worm.

Levamisole has shown *in vitro* activity against adult *T*. *muris* (IC_50_ = 80.8 μM) [8] and *T*. *suis* [53], but it has a poor efficacy against *Trichuris* spp. infections when orally administered to humans [7] and pigs [54, 55]. A plausible explanation for the low *in vivo* efficacy may be a low concentration of levamisole in the large intestine where *Trichuris* spp. are located. This is supported by observations in pigs where the concentrations of levamisole in the large intestine of pigs after oral administration of 7.5 mg/kg levamisole is 1.7–2.6 μg/mL (8.3–12.7 μM) [56], which is below the IC_50_ value (80.8 μM) for adult *T*. *muris* [53]. *Trichuris* spp. are located partly intracellular with the anterior part buried into the mucosa [57] and the posterior part freely moveable in the lumen of the hindgut. Any anthelmintic may reach these worms via the systemic circulation as well by the gastro-intestinal tract. The plasma bioavailability of levamisole in pigs is higher after parental administration than after oral administration [56]. The effect of the route of administration on the efficacy of levamisole has been tested: it was found that parentally administered levamisole results in a higher worm count reduction (WCR = 95.5%) than levamisole given orally (WCR = 40%) [55]. Based on these findings, the low efficacy of levamisole stems from the less favorable pharmacokinetic profile of oral administered levamisole rather than the actual direct effect of the drug.

Monepantel is reported to have no effect on adult *T*. *muris in vitro* and *in vivo* [8]. Pharmacokinetic studies including monepantel and pigs, are to the knowledge of the authors not available, but the intestinal mucosa concentrations of both monepantel and the anthelmintic active metabolite monepantel sulfone [58] have been shown to decrease along the intestinal tract of sheep [59]. A suitable minimum dose of 2.5 mg/kg monepantel was selected by Kaminsky et al. [60] based on the high efficacy of the drug against parasitic nematodes situated in the lower part of the intestine. However, this dose fails to reduce *T*. *ovis* in naturally infected sheep [61].

Derquantel is ineffective against parasitic nematodes of sheep situated in the lower part of the intestine, i.e. *Oesophagostomum* spp. and *Trichuris* spp. [62] which may be related to an extensive absorption of the drug in the first half of the intestinal tract leaving insufficient drug concentrations available to parasites in the hindgut. Absorption in the first half of the intestine is predicted for sheep based on a jird-model and may therefore be different for pigs and humans.

### Treatment strategies for *trichuriasis*

It is likely that in the shorter term, the best strategy for the control of STHs is to use existing anthelmintics in combination therapies rather than to develop new anthelmintic drugs. The rationale is to increase efficacy by using drugs with different modes of actions, to delay the development of anthelmintic resistance and to target several parasitic species (i.e. the STHs) [63]. The efficacy of oxantel pamoate has been evaluated as a monotherapy or in combination with a range of other anthelmintics such as albendazole [21], mebendazole, pyrantel pamoate [64], tribendimidine [65] and moxidectin [66]. For *T*. *trichiura*, the greatest efficacy has been calculated for co-administration of oxantel pamoate and albendazole, whereas the triple combination oxantel pamoate, pyrantel pamoate and albendazole showed the highest efficacy to all the STHs [63]. Another strategy for the control of STHs is to evaluate more recent veterinary anthelmintic drugs such as emodepside [63], which due to its high efficacy against *T*. *vulpis*, even as a single-dose treatment (Profender tablets for dogs) [67], has been approved for the treatment of trichuriasis in dogs [68]. Emodepside has recently shown promising results when tested *in vitro* on *T*. *muris*, and the hookworms *Ancylostoma ceylanicum*, *Necator americanus*, and *in vivo* in animal models infected with the above mentioned parasitic species [69]. Thus, emodepside is a promising drug candidate for the treatment of not only trichuriasis, but for STHs. We point out that the recommendations for treatment of trichuriasis in both human and veterinary medicine, are for the benzimidazoles, administration on 3 consecutive days. [70, 71] However, the 3-day treatment is not compatible with mass drug administration programs for the control of STHs [72] which require single-dose treatments.

In conclusion, the discovery of the *Tsu-O*-AChR provides new insights for the high efficacy and specificity of oxantel on whipworms, and provide us with an example of an anthelmintic, that due to its narrow-spectrum will have a lower impact on non-target nematode species. The advantage of such an anthelmintic is the reduced risk of inducing anthelmintic resistance in other parasitic nematode species, and a lower impact on the environmental biodiversity after drug expulsion from the host (i.e. primarily animal hosts).

## Material and methods

### Ethic statement

The worm material used in this study was obtained during a previous described study [73] performed at the Experimental Animal Unit, University of Copenhagen, Denmark according to the national regulations of the Danish Animal Inspectorate (permission no. 2015-15-0201-00760). The neurologic tissue from *X*. *laevis* was obtained from one adult female, which was anaesthetized by submersion into a tricaine solution (ethyl 3-aminobenzoate methanesulfonate, 2g/L) and subsequently decapitated. All procedures involving live material were performed according to the national regulations of the Danish National Animal Experiments Inspectorate (permission no. 2015-15-0201-00560).

### Drugs

All drugs except 3-bromocytisine, DHβE, α-BTX and derquantel were purchased at Sigma-Aldrich (Copenhagen, DK). 3-bromocytisine, DHβE and α-BTX were obtained from Tocris Bioscience (Abingdon, UK) and derquantel was purchased at Cayman Chemicals (Ann Abor, MI, USA). Stock solutions of drugs were made in either Kulori medium or DMSO (100%) and stored at -20 or 5°C (i.e. ACh) until use. Before use, stock solutions were dissolved in Kulori medium with a maximum final concentration of DMSO of 0.1%.

### Bioinformatics and sequence analysis

The *Asu-*ACR-16 (accession number AKR16139) and the *Cel-*ACR-16 (accession number NP505207) were used as queries in database searches for *Trichuris suis* ACR-16 (*Tsu-*ACR-16) and ACR-16s from other Clade I parasitic nematodes (i.e. *Trichuris* spp. and *Trichinella spiralis*) in the protein-protein BLAST (BLASTp) service at the National Center for Biotechnology Information (NCBI) service [74]. *Cel-*ACR-16 and *Cel-*ACR-19 were used in an alignment with the identified putative ACR-16 sequences from Clade I parasitic nematodes. The accession numbers of the sequences used for the alignment are:

***Caenorhabditis elegans*:** ACR-16 NP_505207, ACR-19 NP_001129756. ***Trichuris suis*:** putative ACR-16 KFD48832.1 putative ACR-19 KFD70086.1. ***Trichuris trichiura***: putative ACR-16 CDW52185; putative ACR-19 CDW53523. ***Trichuris muris***: putative ACR-16 WBGene00290200; putative ACR-19 WBGene00291941. ***Trichuris spiralis***: putative ACR-16 KRY38920.1, putative ACR-19 KRY27533.1.

Signal peptide was predicted using the SignalP 4.1 server [75] and the transmembrane regions were predicted using the TMHMM version 2 server [76]. Deduced amino-acid sequences were aligned using MUSCLE. Phylogenetic analysis was performed on deduced amino-acid sequences. Maximal likelihood phylogeny reconstruction was performed using PhyML V20120412 (https://github.com/stephaneguindon/phyml-downloads/releases) and the significance of internal tree branches was estimated using bootstrap resampling of the dataset 100 times. The accession numbers sequences used for the analysis are:

***Caenorhabditis elegans*:** ACR-7 NP_495647; ACR-8 NP_509745; ACR-9 NP_510285; ACR-10 NP_508692; ACR-11 NP_491906; ACR-12 NP_510262; ACR-14 NP_495716; ACR-15 NP_505206; ACR-16 NP_505207; ACR-19 NP_001129756; EAT-2 NP_496959; LEV-1 NP_001255705; UNC-29 NP_492399; UNC-38 NP_491472; UNC-63 NP_491533.

***Haemonchus contortus***: ACR-16 MH806893. ***Soboliphyme baturini***: acr-16-like VDP07835.1. ***Steinernema glaseri***: ACR-16 KN173365.1. ***Steinernema feltiae***: ACR-19 KN166031.1.

***Toxocara canis***: ACR-16 VDM44142.1; ACR-19 VDM36763.1. ***Parascaris equorum*:** ACR-26 KP756902; ACR-27 KP756903. ***Trichuris suis*:** ACR-16-like KFD48832; ACR-19 KFD70086. ***Trichuris muris*:** ACR-16-like WBGene00290200; ACR-19 WBGene00291941. ***Trichuris trichiura*:** ACR-16-like CDW52185; ACR-19 CDW53523. ***Trichinella spiralis*:** ACR-16-like KRY38920.1; ACR-19 KRY27533.

### cDNA synthesis

Worm material was kept in RNA later (Sigma-Aldrich, Copenhagen, DK) at -20°C until use. Total RNA was extracted from whole adult *T*. *suis* males and females using TRIzol LS Reagent (Invitrogen). For each isolation, 15 worms were used. The RNA was DNAse treated (Invitrogen). The whole brain of one *X*. *laevis* frog was used to extract RNA using Tri Reagent; (MRC. Inc; US) and M tubes in an OctoMacs homogenizer machine (Milteny, Germany) following the manufacturer’ protocol. The isolated RNA was DNAse treated using the RNeasy MinElute Cleanup kit (Qiagen, Germany). The quantity and quality of RNA from *T*. *suis* and *X*. *laevis* was assessed by OD measurement in a Nanodrop spectrophotometer (Thermo Scientific, Demark) and by visual inspection in an agarose gel (1%). First strand cDNA was synthesised from 2.5 μg of RNA from *T*. *suis* whole worms and brain RNA from *X*. *laevis* using SuperScript IV VILO Master Mix (Invitrogen) according to manufacturer’s protocol.

### PCR and cloning of a full-length *T*. *suis ACR-16* subunit and *Xla-RIC-3*

To amplify the full-length coding sequence of the *Tsu-acr-16-like* subunit, we performed a direct PCR using a specific primer pair, containing the *Bam*HI and the *Not*I restriction enzyme sites and 5 additional nucleotides (*Tsu*-*acr-16*-F-5’- GAATC*-Bam*HI-ATGCGGCCGATAATTTTCCTC-3’ and *Tsu-acr-16*-R- 5’-ACGTT *Not*I TCACACAGTTAAATGGGGAGAAC-3’). The specific primers were designed using the putative *Tsu-acr-16-like* sequence in Wormbase under gene number M514_10316 and were designed to target the 5’- and the 3’-end of the putative *Tsu-acr-16-like* cDNA sequence. The specific primer pair used to amplify the *Xla-ric-*3 sequence are described elsewhere [32]. Restriction enzyme sites (*Bam*HI and *Not*I) were included to facilitate ligation into the expression vector pXOOM [77]. PCR amplifications were performed with Platinum SuperFi Green PCR Master Mix (Invitrogen) following the manufacturer’ recommendations. Amplicons were evaluated by gel electrophoresis, purified with QIAquick Gel Extraction Kit (Qiagen), cloned into pXOOM and sequenced. A positive clone of *Tsu-acr-16-like* subunit and *Xla-ric-3* was selected and linearized with the restriction enzyme *Nhe*I before *in vitro* transcription using the mMessage mMachine T7 Transcription Kit (Ambion). In parallel, cRNAs for the RIC-3 homologs from *A*. *suum*, *H*. *contortus* and *C*. *elegans* were also synthesized. The cRNAs were purified using MEGAclear (Thermo Scientific, Demark). Quantity and quality of cRNAs was evaluated by OD measurement in a Nanodrop spectrophotometer (Thermo Scientific, Demark) and by visual inspection in an agarose gel (1%).

### Microinjection of *Xenopus laevis* oocytes

*Xenopus laevis* oocytes were obtained from EcoCyte Bioscience (Castrop-Rauxel, Germany) and kept at 19°C in Kulori medium (90 mM NaCl, 4 mM KCl, 1 mM MgCl_2_, 1 mM CaCl_2_, 5 mM HEPES, pH:7.4) until injection. Oocytes were injected with 50 nl of cRNA in RNAse-free water using a microinjector (Nanojet, Drummond Broomal, PA, USA). To test *ric*-3 effects on the receptor expression, 25 ng *Tsu-acr-16-like* cRNA was injected alone or with 5 ng of either of the following ric-3 cRNAs: *Asu*-*ric*-3, *Xla*-*ric*-3, *Hco-ric*-3.1 or *Cel-ric*-3. To exclude endogenous nAChR expression induced by *Asu*-*ric*-3, 5 ng *Asu*-*ric*-3 was injected alone. These amounts of cRNA were chosen in order to compare the drug-potency results of the *Tsu-*ACR-16-like receptor with that of *Asu-*ACR-16[29]. Only half (i.e. 12.5 ng *Tsu-acr-16-like* cRNA and 2.5 ng *Asu*-*ric*-3) was used for the dose-response curves and antagonist analysis. To allow for receptor expression, the injected oocytes were incubated in Kulori medium at 19°C for 3–7 days and the Kulori medium was changed daily.

### Two-electrode voltage clamp of *Xenopus laevis* oocytes

Two-electrode voltage-clamp (TEVC) recordings were obtained using an Oocyte Clamp Amplifier OC-725 B (Warner Instruments Corp., USA) connected to an Axon Digidata 1440A digitizer (Axon Instruments, Molecular Devices, USA) and was performed at ~19°C under continuous flow of Kulori medium with the oocytes clamped at -60 mV. Data were sampled at 2 kHz using the pClamp 10.4 acquisition software (Axon Instruments, Molecular Devices, USA). The microelectrodes were pulled from glass capillaries (TW 120.3, World precision instruments, USA) on a programmable micropipette puller (Narishige, Japan). The resistance when filled with 3 M KCl ranged from 0.5 to 1.5 MΩ. Ag/AgCl reference electrodes were connected to the bath with agar bridges. For a minimum of 4 hours prior to recording, all oocytes were incubated in BAPTA-AM at a final concentration of 100 μM to chelate intracellular Ca^2+^ ions and hereby prevent activation of endogenous calcium activated chloride channels during recordings. For each experiment, 6 positive- and 6 negative control oocytes were exposed to 100 μM ACh for 10 s on the day of each experimentation. Recordings of oocytes co-injected with *Tsu-acr-*16-like and *Asu-ric*-3 cRNAs were used as positive controls, and recording of non-injected oocytes as negative controls. The quality of each oocyte was evaluated before drug exposure, i.e. only injected oocytes with a membrane potential between -40 to -60 mV before clamping and a current injection below 0.1 μA after clamping were included in the experiments. To determine the reversal potential for the activated receptor in presence of low (1 mM) and high (10 mM) CaCl_2_ concentrations, the oocytes were exposed to 100 μM ACh and subsequently currents were measured at voltage between– 40 mV to 40 mV (10 mV increments). We cannot exclude that some oocytes exposed to non-activating drugs were poorly expressing oocytes as we did not apply ACh after the test drug exposure. However, with the number of oocytes tested from at least 3 batches of eggs, we find it most unlikely that this would have an impact on the overall results.

### Drug-potency-tests

All agonists were used at a final concentration of 100 μM and were tested in 3–4 experiments using 3–4 different batches of oocytes batches. For each experiment, 6 oocytes were tested per drug. For each experiment, 6 oocytes were exposed to 100 μM ACh for 10 s, and all other responses in the same experiment, were normalized to the mean response of these controls. As the ACR-16 of the parasitic nematode *A*. *suum* has been reported to respond to a 10 s application period of oxantel [29], we applied the same application period in our studies for comparison. Initial experiments showed a consistent decrease in the response to 100 μM ACh when applied repeatedly, even after washing periods for up to 5 min between agonist applications. An example of this can be seen in Fig 7B where the same oocytes were exposed to 100 μM ACh 3 times separated by washing periods of 5 min. Therefore, each drug was tested on oocytes not previously exposed to ACh (100 μM). The total number of oocytes examined per drug was: *n* = 23 for oxantel, *n* = 16 for pyrantel, *n* = 15 for epibatidine, *n* = 16 for nicotine, *n* = 15 for 3- bromocytisine, *n* = 16 for DMPP, *n* = 17 for morantel, *n* = 17 for cytisine and *n* = 15 for levamisole. Each drug was applied for 10 s followed by wash off until the current had returned to pre-stimulation values.

### Dose-response studies

The dose-response studies were in total performed on 3 different oocyte batches. For acetylcholine the number of measurements per drug concentration were 9. For oxantel the number of measurements per drug concentration were as follows: 0.3 μM, *n* = 6; 0.1 μM, *n* = 6; 3 μM, *n* = 6; 3 μM, *n* = 6; 10 μM, *n* = 15; 30 μM, *n* = 12; 100 μM, *n* = 13; 300 μM, *n* = 14. For pyrantel *n* per drug concentration were: 1 μM, *n* = 6; 3 μM, *n* = 6; 10 μM, *n* = 6; 30 μM, *n* = 6; 100 μM, *n* = 15; 300 μM, *n* = 23; 1000 μM, *n* = 12; 3000 μM, *n* = 20. For each experiment, 6 oocytes were exposed to 300 μM ACh for 10 s, and all other responses in the same experiment, were normalized to the mean response of these positive controls. The ACh concentration of 300 μM was used to reach the maximal activation of the *Tsu-*ACR-16-like receptor. Each drug and drug concentration were tested as described for the drug-potency-tests.

### Antagonists

The effects of the antagonists DHβE, derquantel and α-BTX (10 μM) were examined in the presence 100 μM ACh as previously described for *Asu-*ACR-16 [29]. In short, *X*. *laevis* oocytes co-injected with *Tsu-acr-16-like-* and *Xla-ric*-3 cRNA were sequentially superfused with ACh for 10 s, then ACh + antagonist for 10 s, and finally with ACh for 10 s. For α-BTX a five-step protocol including a pre-incubation (10 s) with the antagonist (10 μM) was used with ACh (100 μM). *Xenopus laevis* oocytes, co-injected with *Tsu-acr-16-like-* and *Xla-ric*-3 cRNAs, were exposed to: i) a control application of 100 μM ACh for 10 s (first application); ii) followed by a wash-off period of 5 min; iii) then by an application of 10 μM α-BTX for 10 s, immediately followed by 100 μM ACh and the continued presence of α-BTX for 10 s (second application); iv) then a wash-off period of 5 min; v) and finally an application of ACh for 10 s (third application). Control oocytes were exposed to ACh for 10 s in 3 consecutive steps, each separated by a wash-off period of 5 min. For each antagonist, *n* = 6–8.

### Electrophysiological data and statistically analysis

All acquired electrophysiological data were analysed with Clampfit 10.7 (Molecular Devices, Sunnyvale, CA, USA) and GraphPad Prism 8 (GraphPad Software, La Jolla, CA, USA) and from all experiments, peak currents from BAPTA-AM-incubated oocytes were measured after application of drugs. For the auxiliary protein (RIC-3) test, the group mean current of oocytes co-injected with *Xla-ric*-3 and *Tsu-acr*-16-like cRNAs in response to 100 μM ACh was set to 100%, and all other responses were normalized to this. The relative means were statistical analysed using a non-parametric Kruskal-Wallis Test and *P* < 0.05 was considered significant. For the drug-potency-test, peak currents of drugs were normalized to the peak current measured in the presence of 100 μM ACh and was expressed as mean ± SEM. Data was tested for normality using the D’Agostino-Pearson normality test. Drug-group means were statistical analysed using One-Way ANOVA with a Turkey’s Multiple Comparison Test where *P* < 0.05 was considered significant.

For the dose-response relationships, and for each experiment, 6 oocytes were exposed to ACh (300 μM) for 10 s, and all other drug responses in the same experiment, were normalized to the mean response of these controls. The normalized current as a function of drug concentration allowed fitting the dose-response curves with a Hill equation, using nonlinear regression analysis with a variable slope model in GraphPad Prism 8. The following equation was used:

Irel=Imin+(Imax–Imin)/(1+10^((LogEC50–[D])*nH)),

where *I*_rel_ is the mean relative current, *I*_max_, is the relative current obtained at saturating agonist concentration, *I*_min_ is the relative current obtained at agonist concentration 0 μM, *EC*_*50*_ is the concentration of agonist resulting in 50% of the maximal current response, [*D*] is the drug concentration and *n*_H_ is the Hill coefficient. *I*_max,_ *EC*_*50*_ and *n*_H_ were fitted as free parameters whereas *I*_min,_ was constrained to 0.

For the antagonist test with α-BTX, the current response of α-BTX and ACh in the continued presence of α-BTX (second application) was normalized to the first response at 100 μM ACh (first application) which was set to 1. The group mean of the α-BTX response were statistical analysed using One-Way ANOVA with a Dunnett’s test.

### Caenorhabditis elegans experiments

Wildtype worms N2 were used to express the *Tsu-*ACR-16-like receptor under the control of the myosin promotor present in the plasmid *pmyo3*, pPD96.52 (Addgen). The *pmyo3* plasmid was co-injected at 60 ng/μL with plasmid pPD118.33_*pmyo2*::*gfp* (Addgen) at 30 ng/μL into the gonads of young adult hermaphrodites worms as previously described [78]. The pPD118.33_*pmyo2*::*gfp* was used as a transformation marker and three stable recombinant *C*. *elegans* lines were generated. The motility of all lines was determined by counting the thrashes of individual gravid adult worms for 30 s. All thrashing assays were performed in 96-well plates containing M9 buffer with 0.1% BSA, either with or without 500 μM of oxantel. The basal motility of wt N2 and each of the recombinant lines was establish after 10 min equilibration time in M9 buffer. The effect of oxantel on the worms was evaluated after 24 h incubation in M9 buffer with 500 μM of oxantel. Wild type N2 and each of the recombinant lines not exposed to 500 μM oxantel for 24 h were used as negative controls. The motility of each line was statistically analyzed using Kruskal-Wallis- and Wilcoxon’s test.

## Supporting information

S1 Fig Distance tree showing relationships of the ACR-16-like acetylcholine receptor (AChR) subunits from Clade I nematode species, with other AChR subunits from the ACR-16 group from nematode species representative from Clade III, Clade IV, and Clade V. NJ-Tree was built upon an alignment of AChR subunit deduced amino-acid sequences. The tree was rooted with the *C*. *elegans* UNC-63 sequence. Scale bar represents the number of substitutions per site. Bootstrap values (1000 replicates) are indicated on branches. Accession numbers for sequences used in the analysis are provided in Material and Methods section. Nematode clades refer to Blaxter et al. 1998 [1]. AChR subunit sequences from Clade I species are highlighted in red, AChR subunit sequences from Clade III species are highlighted in blue, AChR subunit sequences from Clade V species are highlighted in pink (in black for *C*. *elegans*). *Cel*, *Hco*, *Sba*, *Sgl*, *Sfe*, *Tca*, *Tsp*, *Tsu*, *Ttr* and *Tmu* refer to: *Caenorhabditis elegans*, *Haemonchus contortus*, *Soboliphyme baturini*, *Steinernema glaseri*, *Steinernema feltiae*, *Toxocara canis*, *Trichinella spiralis*, *Trichuris suis*, *Trichuris trichiura*, and *Trichuris muris* respectively.
(TIF)

S2 Fig Desensitization kinetics of *Tsu-*ACR16-like receptor. A representative response of the *Tsu-*ACR16-like receptor to 1 min exposure of 100 μM ACh. The *Tsu-*ACR16-like receptor is characterized by a slow-desensitization kinetic as compared to *Asu*-ACR-16 [29] and *Peq*-ACR-16 [33].
(TIF)

S3 Fig Viability of N2 and recombinant *Caenorhabditis elegans* lines after 24 hours. Boxplot depicts number of thrashes/30 sec of worms in M9 after 24 h. The number of thrashes were not significant between neither of the lines when oxantel was not included in the M9 buffer.
(TIF)

S1 Video The movie shows the motility of adult wild type N2 (absence of a green fluorescent pharynx) and recombinant *Caenorhabditis elegans* harbouring the *Tsu-acr*-*16-like* receptor (green fluorescent pharynx) after 24 hours incubation in M9 buffer with 500 μM oxantel. Note the lower motility of the recombinant worms.
(MP4)
