# Supplementary figures and images for: The narrow-spectrum anthelmintic oxantel is a potent agonist of a novel acetylcholine receptor subtype in whipworms
Source: PLoS Pathog. 2021 Feb 5;17(2):e1008982. doi: 10.1371/journal.ppat.1008982 (PMC7891710; doi:10.1371/journal.ppat.1008982)

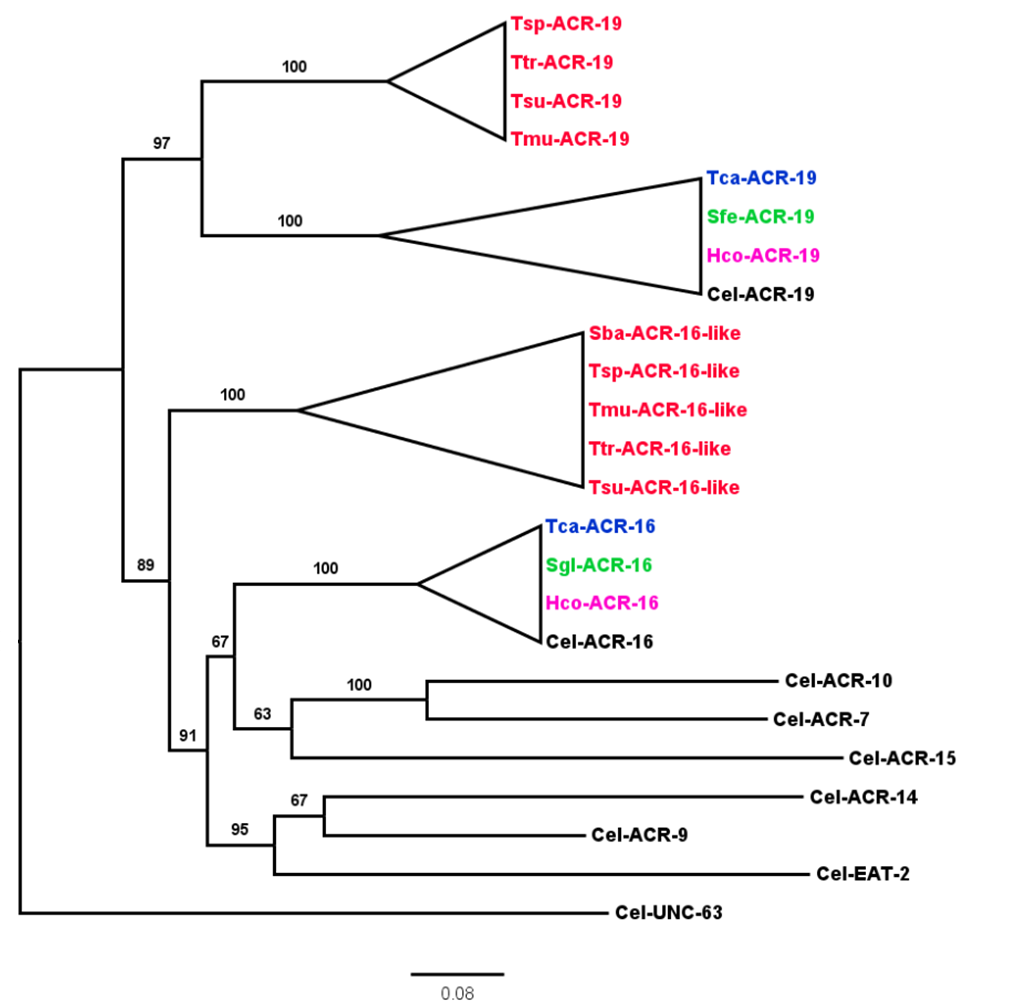

Supplement: S1 Fig — NJ-Tree was built upon an alignment of AChR subunit deduced amino-acid sequences. The tree was rooted with the C. elegans UNC-63 sequence. Scale bar represents the number of substitutions per site. Bootstrap values (1000 replicates) are indicated on branches. Accession numbers for sequences used in the analysis are provided in Material and Methods section. Nematode clades refer to Blaxter et al. 1998 [1]. AChR subunit sequences from Clade I species are highlighted in red, AChR subunit sequences from Clade III species are highlighted in blue, AChR subunit sequences from Clade V species are highlighted in pink (in black for C. elegans). Cel, Hco, Sba, Sgl, Sfe, Tca, Tsp, Tsu, Ttr and Tmu refer to: Caenorhabditis elegans, Haemonchus contortus, Soboliphyme baturini, Steinernema glaseri, Steinernema feltiae, Toxocara canis, Trichinella spiralis, Trichuris suis, Trichuris trichiura, and Trichuris muris respectively. (TIF) [file ppat.1008982.s001.tif]

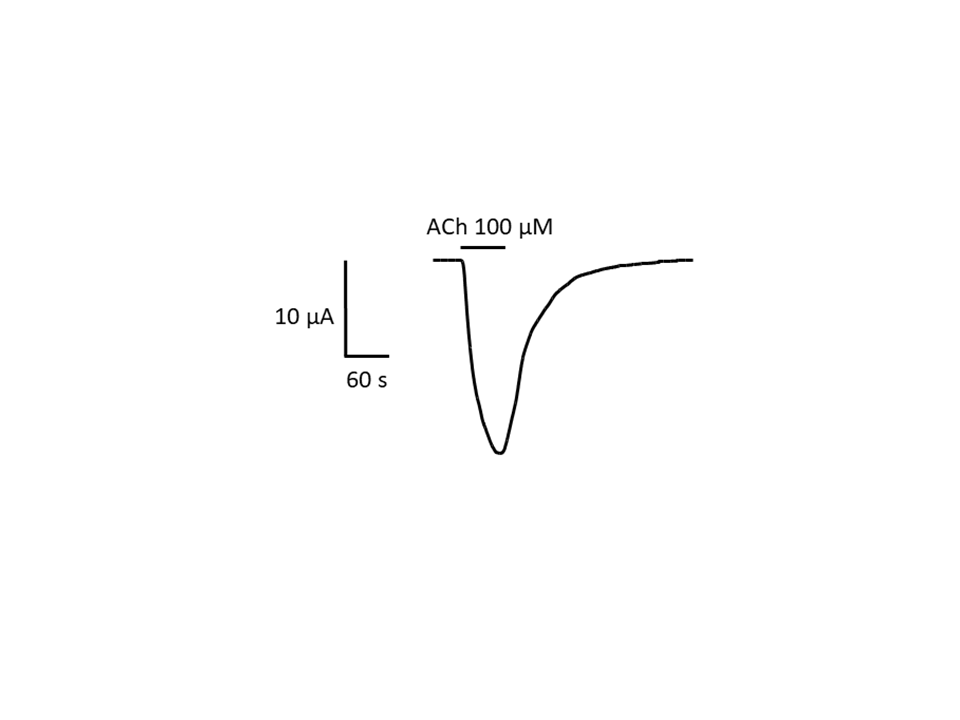

Supplement: S2 Fig — A representative response of the Tsu-ACR16-like receptor to 1 min exposure of 100 μM ACh. The Tsu-ACR16-like receptor is characterized by a slow-desensitization kinetic as compared to Asu-ACR-16 [29] and Peq-ACR-16 [33]. (TIF) [file ppat.1008982.s002.tif]

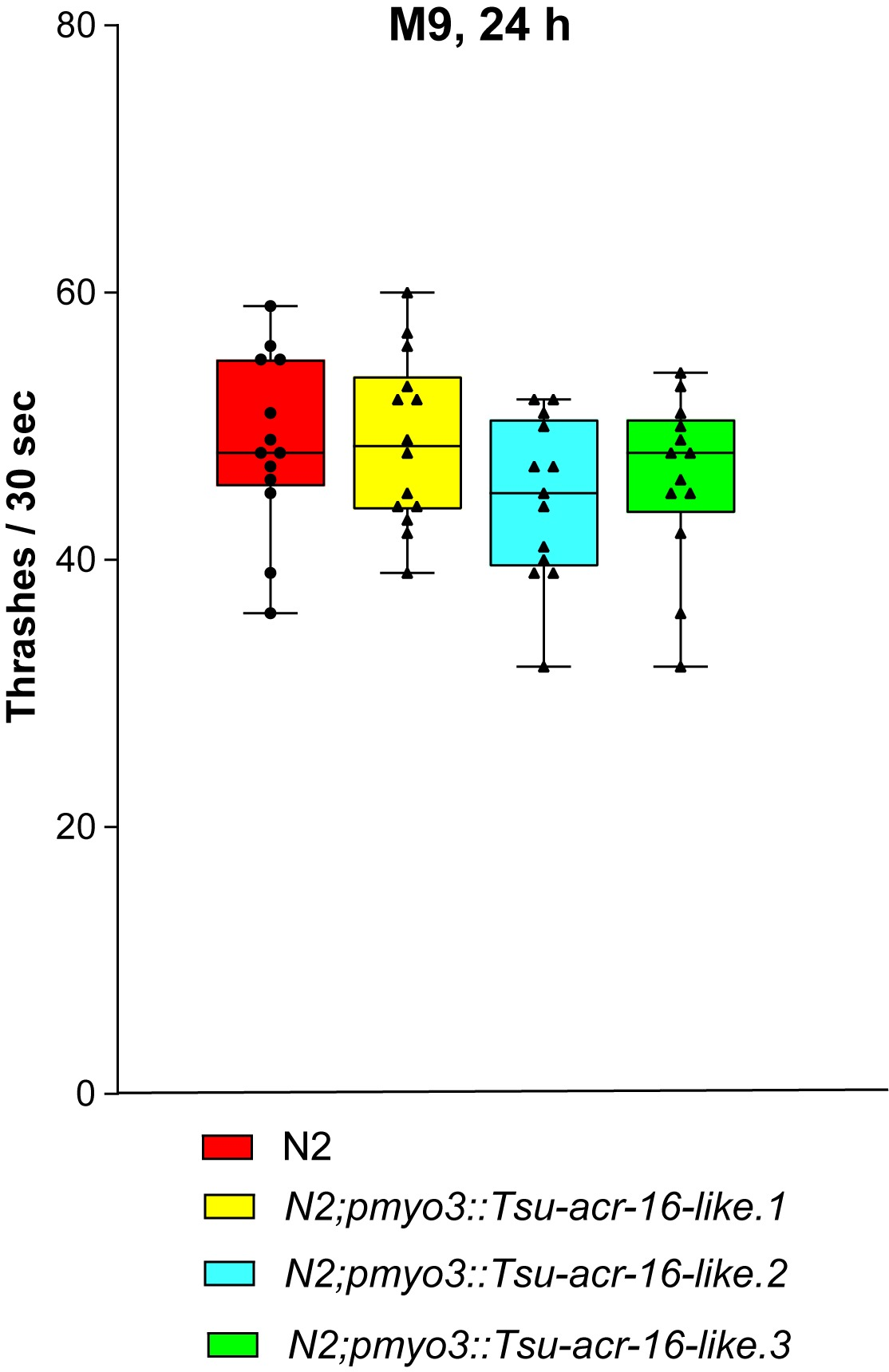

Supplement: S3 Fig — Boxplot depicts number of thrashes/30 sec of worms in M9 after 24 h. The number of thrashes were not significant between neither of the lines when oxantel was not included in the M9 buffer. (TIF) [file ppat.1008982.s003.tif]
